# Cold Temperatures Increase Cold Hardiness in the Next Generation *Ophraella communa* Beetles

**DOI:** 10.1371/journal.pone.0074760

**Published:** 2013-09-30

**Authors:** Zhong-Shi Zhou, Sergio Rasmann, Min Li, Jian-Ying Guo, Hong-Song Chen, Fang-Hao Wan

**Affiliations:** 1 State Key Laboratory for Biology of Plant Diseases and Insect Pests, Institute of Plant Protection, Chinese Academy of Agricultural Sciences, Beijing, China; 2 Ecology and Evolutionary Biology, University of California Irvine, Irvine, California, United States of America; 3 Fujian Entry-Exit Inspection and Quarantine Bureau, Fuzhou, China; University of Lausanne, Switzerland

## Abstract

The leaf beetle, *Ophraella communa,* has been introduced to control the spread of the common ragweed, *Ambrosia artemisiifolia,* in China. We hypothesized that the beetle, to be able to track host-range expansion into colder climates, can phenotypically adapt to cold temperatures across generations. Therefore, we questioned whether parental experience of colder temperatures increases cold tolerance of the progeny. Specifically, we studied the demography, including development, fecundity, and survival, as well as physiological traits, including supercooling point (SCP), water content, and glycerol content of *O. communa* progeny whose parents were maintained at different temperature regimes. Overall, the entire immature stage decreased survival of about 0.2%–4.2% when parents experienced cold temperatures compared to control individuals obtained from parents raised at room temperature. However, intrinsic capacity for increase (*r*), net reproductive rate (*R*_0_) and finite rate of increase (*λ*) of progeny *O. communa* were maximum when parents experienced cold temperatures. Glycerol contents of both female and male in progeny was significantly higher when maternal and paternal adults were cold acclimated as compared to other treatments. This resulted in the supercooling point of the progeny adults being significantly lower compared to beetles emerging from parents that experienced room temperatures. These results suggest that cold hardiness of *O. communa* can be promoted by cold acclimation in previous generation, and it might counter-balance reduced survival in the next generation, especially when insects are tracking their host-plants into colder climates.

## Introduction

In a changing environment, such as during range expansion, organismal rapid adaptation to novel environmental conditions is needed for assuring survival [1]. If the new environment a parent is experiencing predicts the characteristics of the progeny’s environment, then parents may enhance their net reproductive success by differentially allocating resources to their offspring so to create novel phenotypes more adapted to the novel environment. Such maternal effects have been reported in both animals and plants [2], [3], [4], [5], [6]. For example, an insect mother’s experience of environmental changes such as photoperiod, temperature and nutrition have been shown to impact several physiological and ecological traits [2], [3], [4], [5], [7], [8], as well as improve fitness related traits (e.g. development and fecundity) of the offspring [2], [3], [4], [9]. Many previous studies have shown that insect’s growth and population development in progeny were influenced by their mother’s experiences of temperature changes [10], [11], [12], [13], [14], [15], [16], [17], [18], [19], [20], [21], [22]. For example, repeated exposition to cold temperatures in female flies resulted in a change in sex ratio and number of offspring [21], [22].

Because of insects’ ectothermic physiology, temperature, among other environmental factors, is a key factor affecting individual survival, fecundity and population establishment in the field [10], [16], [23], [24], [25], [26], [27]. Insects may be significantly impacted if temperature deviates from the optimum range [18], however, the amplitude of the impact depends on the rate, intensity, and duration of temperature change [28], [29]. Resistance to cold temperatures, or cold hardiness of insects, is reflected by four variables relating to insect physiology including; water content, accumulation of glycerol or other low molecular weight polyols and sugars (cryoprotectants), supercooling point (SCP), and survival capacity under low temperature [28], [30], [31], [32], [33], [34]. Previous studies revealed that better cold hardiness is typically associated with lower SCP, lower water content, and higher glycerol content in the insect [28], [30], [31], [35], [36], [37], [38], [39], [40].

Maternal effects result in alterations of the offspring phenotype [2], [3], [5], in which, biological and ecological adaptability of offspring can be related to mother’s experience [4], [7], [8], [41]. We can thus posit that temperature-driven maternal effects are an excellent strategy for insect herbivores to be able to track range expansion of the host plant into colder climates. Therefore, we here specifically hypothesized that mother’s experience of cold temperatures can pass on to the offspring, promoting faster development, higher fecundity and higher cold hardiness in the next generation. We tested this hypothesis using *Ophraella communa* (Coleoptera: Chrysomelidae). Adults and larvae of the beetle feed on leaves of the common ragweed *Ambrosia artemisiifolia* [42], [43], [44]. Due to the insect potential to drastically reduce plant fecundity [45], [46], [47], *O. communa* is currently considered as biological control agent for reducing the spreading of the common ragweed. However, insects may be disfavored when common ragweed populations are expanding into colder regions of China [48]. This may provide the plant an “enemy-free space” [30], [35]. Indeed, previous study revealed that the development and female fecundity of *O. communa* were affected after 2 hours of low temperature stresses [17]. However, during the gradual temperature decrease from summer to winter, cold hardiness of *O. communa* is progressively increased [37]. Adults overwinters in soil and can even survive severe subzero temperatures during winter [43]. Therefore, if maternal effects are adaptive [49], we should expect cold hardiness of the progeny being enhanced by maternal and paternal cold experience. We measured fitness parameters (survival rate, longevity, fecundity, and life table analyses) and cold hardiness indices (SCP, water and glycerol contents) of *O. communa* offspring originated from parents that experienced different temperature regimes for several days.

## Materials and Methods

### Host Plants and Insects

The common ragweed, *Ambrosia artemisiifolia* is the most widespread plant of the genus *Ambrosia* in North America and has become invasive in most European as well as Asian countries such as Japan and China [48]. Seeds were harvested in Hunan province, China, and seedlings, grown in nurseries, were individually transplanted into plastic pots (Φ15 cm) with uniform loamy clay soil from vegetable field. Potted plants were kept in greenhouse under 26–29°C, 65±5% RH, and L14:D10 photoperiod, watered once every four days, and fertilized (N:P:K at the ratio of 13∶7:15) twice a month. Plants were used for experiments when they reached a height of about 50 cm.

*Ophraella communa* pupae were collected from *A. artemisiifolia* plants in Miluo county (28°49.089′N, 113°03.876′E), Hunan province, China, and then stored in a transparent plastic box (19 cm × 12 cm × 6 cm) covered with organdy mesh fabric in an insectary at 28°C, 70±5% RH, and L14:D10 photoperiod. An initial double pair of adults per plant was let freely reproduce, and after the fourth generation of continuous rearing, newly emerged, unmated adults were separated according to gender (n = 20 adults per plant) for two days before being randomly assigned to the different treatments (see below).

Both *A. artemisiifolia* and *O. communa* are not protected in China, thus no specific permissions were required for these locations/activities (e.g, the authority responsible for a national park or other protected area of land, the relevant regulatory body concerned with protection of wildlife, etc.).

### Transgenerational Temperature Effect on *O. communa* Development, Survivorship, and Fecundity

Previous studies have shown that minimum threshold temperatures of egg, larva, pupa and entire immature stage of *O. communa* were 14.1°C, 16.9°C, 9.0°C and 13.3°C, respectively [42], and female fecundity decreased by 26.4% to 70.4% after pupal cold-storage at 4–12°C for 20 days [50]. Based on these findings, the present experiment was designed as follows: 30 females and 30 males (kept separated) were put in similar plastic boxes as above, and transferred randomly in three environmental chambers (PRY-450D, Ningbo Haishu Aifu Experimental Equipment Co. Ltd., Zhejiang, China). Each chamber was set with identical environmental parameters (humidity 70±5% RH, photoperiod L14:D10) except for temperatures that were set at 8, 6, 4°C in each chamber respectively. Individuals in the control group were kept in the insectary under same light and humidity conditions as above but at a temperature of 28°C. Thus, the experiment included four temperature treatments, resulting in a total number of beetles used = 30 individuals * 2 sexes * 4 treatments = 240 adults. After eight days at different temperatures, out of the 30 males and 30 females in each box, 6 males and 6 females were randomly chosen and placed by pair on individual potted plants to obtain 80 experimental eggs (the F_1_ generation/or progeny) per treatment (eggs were randomly collected from each pair resulting in 13–15 eggs per female/treatment). This whole procedure for obtaining the 80 experimental eggs was repeated 5 times to generate the independent replicates for each treatment (n = 5 replicates/treatment). For each treatment, all 80 eggs that were less than 12 hrs-old were placed in plastic basin (50×30 cm) and let emerge to obtain the larval stage. Larvae were maintained on *A. artemisiifolia* plants until pupation. Pupae were then detached from leaves, placed in an unsealed cuvette individually, and monitored daily until adult emergence. Survival rates and developmental period of the different developmental stages (egg, larval, pupae, and entire immature stage) were recorded.

Newly emerging adults were randomly sampled and paired by sex, and provided daily with fresh *A*. *artemisiifolia* leaves as oviposition substrate (N = 25 adult pairs per treatment and per replicate). All eggs laid on twigs were counted until the female died. Longevity of adult beetles was finally recorded.

### Transgenerational Temperature Effect on Physiological Parameters Related to Cold Hardiness

To assess insect’s water content of progeny, 10 F_1_ adults of similar size and weight were randomly chosen from the treatments described above. Each beetle was weighed on an electronic balance (AB204-S, sensitivity ≤0.1 mg, Mettler Toledo, Greifensee, Switzerland) to determine its fresh weight (FW) before being placed in an oven at 70°C for 48 h and reweighed individually to obtain dry weight (DW), and then water content (WC) calculated as WC = (FW – DW)/FW×100%.

For measuring SCP, an additional 30 F_1_ adult beetles from each treatment were removed from ragweed plants and then starved for 12 h. Each adult beetle abdomen was fixed to a thermocouple that was attached to an automatic data recorder (uR100, Model 4152, Yokogawa Electric Co., Seoul, Korea) via a bridge. The thermocouple with the adult was then lowered into a freezing chamber at −25°C and the body temperature of the adult beetle was monitored as it decreased at a rate of about 1°C per minute from 28°C [30]. The SCP was taken to be the temperature recorded by the thermocouple just before the sudden increase in temperature caused by the emission of the latent heat of crystallization [51].

Finally, glycerol was measured from 5 male and 5 female F1 adult beetles from each treatment. The whole-body glycerol content of beetles was determined using an enzymatic assay (337-40A, Sigma Chemical Company, St. Louis, MO, USA) as in [38]. Briefly, twelve hours-starved individual adults were homogenized in 25 mM sodium phosphate buffer (pH 7.4) and then centrifuged at 12,000*** g*** for 10 min at 25°C. The supernatant was then deproteinized with 6% (w/v) perchloric acid, and the protein precipitate that formed was removed by centrifugation (12,000*** g*** for 5 min). The supernatant was then neutralized with 5 M potassium carbonate to pH 7.0. Glycerol levels were determined spectrophotometrically by measuring sample absorbance at 540 nm [52].

### Statistical Analyses

We conducted one-way ANOVAs for testing temperature effect on survival rates and developmental durations of eggs, larvae, pupae, and the entire immature stage, as well as for the testing treatment effect on life-table parameters estimated as follow: 1) net reproductive rate 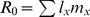; 2) mean generation time 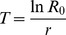; 3) finite rate of increase *λ* = exp(*r*); where *x* is the age in days of the beetle; *l_x_* is the age-specific survival rate; *m_x_* is age-specific fecundity, and 4) the intrinsic rate of increase (*r*) estimated by using the Euler-Lotka formula 
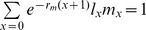
 with age indexed from 0 [53], [54], [55]. Overall, if stage differentiation was ignored, a single age-specific survival rate (*l_x_*) curve of progeny *O. communa* gave the probability that an egg will survive to age *x* in all treatments. The computer program TWOSEXMSChart was used to analyze the life history raw data [54], [56].

Two-way ANOVAs were conducted for testing the interactive effects of temperature, and gender on the survival rates, longevities and cold hardiness physiological parameters of *O. communa* adults. Prior to analyses, the developmental duration and the survival rates of insects were log10(x+1) and arcsine square-root-transformed, respectively, to meet normality assumptions. Student’s post-hoc analyses were performed to measure treatment differences [57].

## Results

### Transgenerational Temperature Effect on *O. communa* Life-history Traits

#### Immature stage

**Figure 1:**
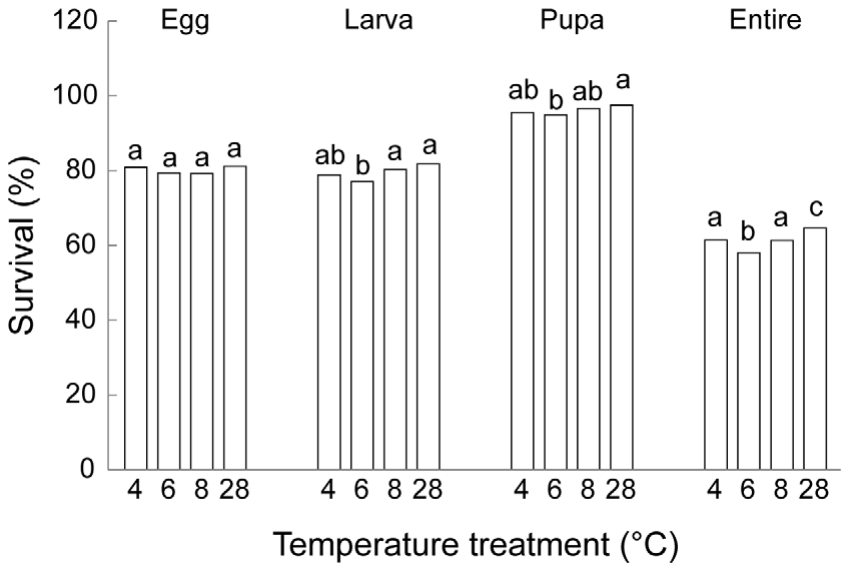
Transgenerational cold treatment induction on next generation *O. communa* immature stages survival (shown is average±1SE). Parent beetles experienced cold temperatures (4, 6, 8°C), or normal room temperatures of 28°C for eight days prior mating. Different letters above bars mean significant differences (*P*<0.05, Tukey HSD post-hoc test).

Overall, temperature experienced by parents did not affect development time of immature stages in the next generation (Table 1). However, temperature treatment strongly affected survival (Table 1, Fig. 1). In particular, the overall immature stage survived 8% worst when parents were placed at cold temperatures when compared to control 28°C (Fig. 1).

**Table 1:** One-way ANOVAs for temperature treatment effect on *O. communa* development time and survival of each immature stage of the beetle life cycle (egg, larva, and pupa) and the entire immature stage.

Response variable	Life stage	*F*	*P*
Development time	Egg	1.67	0.213
	Larva	0.02	0.995
	Pupa	1.33	0.298
	Entire immature stage	0.46	0.716
Survival	Egg	2.51	0.096°
	Larva	7.22	0.003**
	Pupa	3.41	0.043*
	Entire immature stage	17.35	<0.0001***

*** p<0.001,

** p<0.01,

* p<0.05,

°p<0.1.

Temperature treatments are 4, 6, 8, and 28°C, df = 3,16.

#### Adults

**Figure 2:**
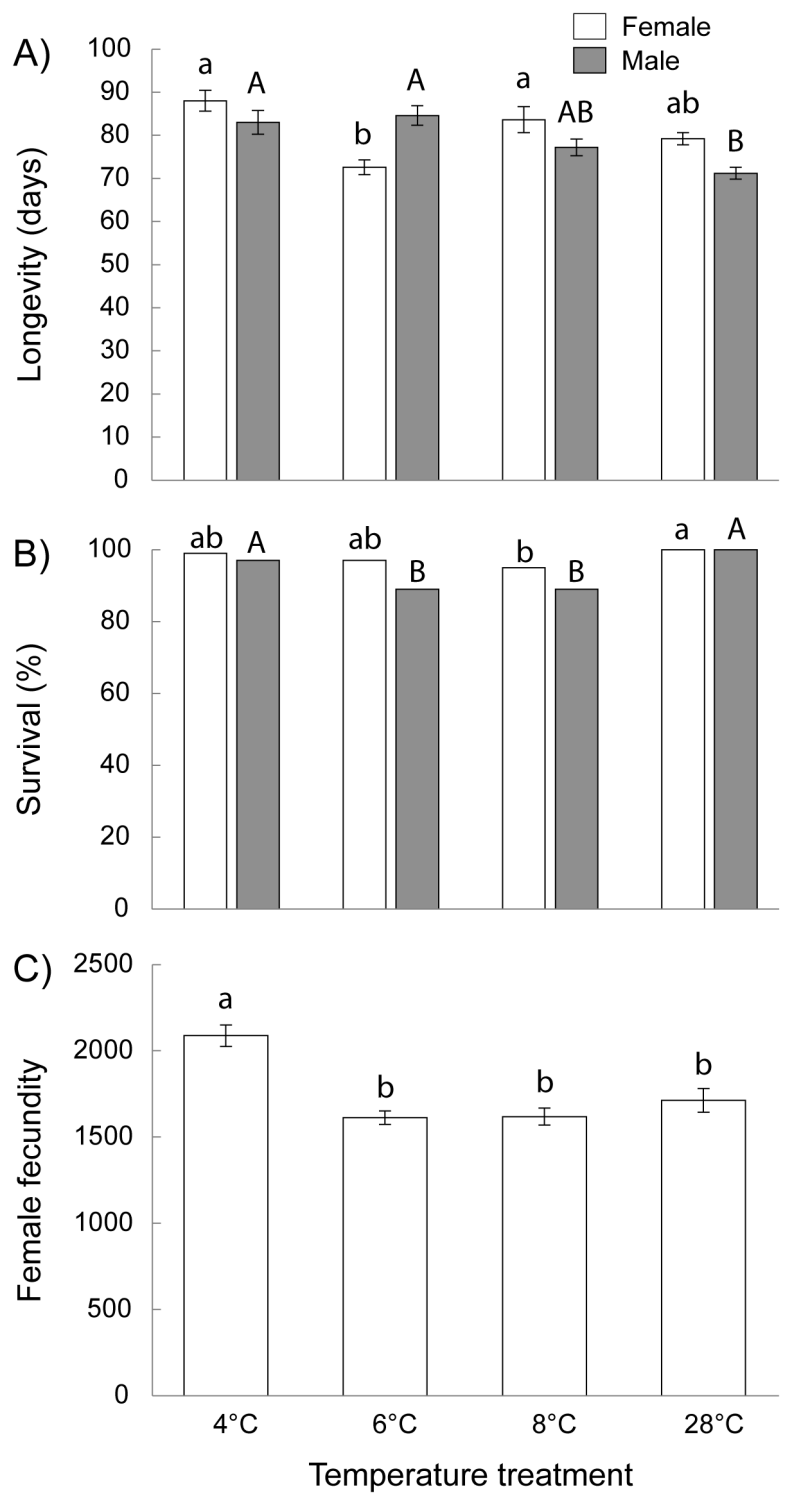
Transgenerational cold treatment induction on next generation *O. communa* A) development time, B) survival, and C) female fecundity (shown is average±1SE). Parent beetles experienced cold temperatures (4, 6, 8°C), or normal room temperatures of 28°C for eight days prior mating. Different letters above bars mean significant differences (*P*<0.05, Tukey HSD post-hoc test).

After cold temperature treatment, adults in the next generation lived longer, with in average a 13% difference in longevity between the 4°C and the 28°C treatment (Table 2, Fig. 2A). Also, all adults descending from parents that experienced 28°C before mating survived (Fig. 2B). We also found temperature variation effect of parent beetles on the next generation female fecundity (Fig. 2C, *F*_3,16_ = 16.20, *P*<0.0001), with parents experiencing 4°C producing 21% more fecund females when compared to the average of the other treatments.

**Table 2:** Two-way ANOVAs for effects of temperature treatment, and gender on *O.communa* next generation adult survival and longevity.

Response variable	Factor	Num. df	*F*	*P*
Survival	Gender (G)	1	19.85	<0.0001***
	Temperature (T)	3	18.50	<0.0001***
	G*T	3	4.13	0.014**
Development time	G	1	1.43	0.241
	T	3	7.71	0.001***
	G*T	3	9.06	0.0002**

*** p<0.001,

** p<0.01,

* p<0.05,

°p<0.1.

Temperature treatments are 4, 6, 8, and 28°C, den. Df = 16.

#### Life-table parameters

Parents’ cold temperature experience significantly affected the net reproductive rates (*R*_0_) (*F*_3,16_ = 4254.35, *P*<0.0001), mean generation time (*T*) (*F*_3,16_ = 13.80, *P*<0.0001), finite increase rates (*λ*) (*F*_3,16_ = 16.36, *P*<0.0001), and intrinsic increase capacities (*r*) (*F*_3,16_ = 70.86, *P*<0.0001) of the progeny. Maximum *r*, *R*_0 and_ *λ*-values were observed when parents experienced 4°C eight days of cold treatments. Mean generation time (T) had more idiosyncratic responses, with a maxima value when parents experienced 6°C.

### Transgenerational Temperature Effect on *O. communa* Cold Hardiness and Physiological Traits

**Figure 3:**
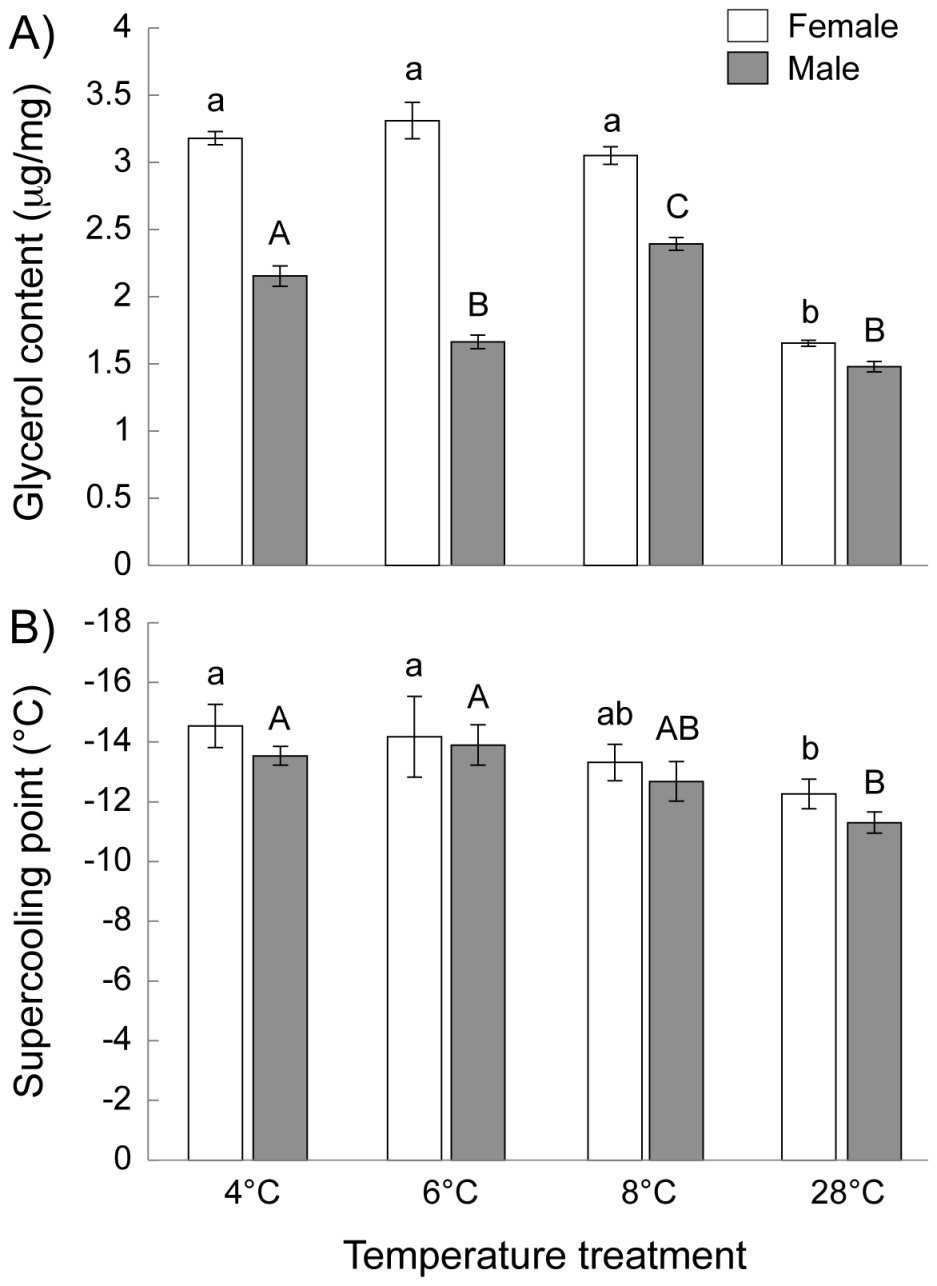
Transgenerational cold treatment induction of increased cold hardiness in *O. communa*. Shown is A) average (±1SE) glycerol content, and B) supercooling point (SCP) of females (open bars) and males (grey bars) beetles when parents experienced cold temperatures (4, 6, 8°C), or normal room temperatures of 28°C. Different letters above bars mean significant differences (*P*<0.05, Tukey HSD post-hoc test).

Parents experiencing cold temperatures influenced next generation adults’ physiological traits by increasing overall cold hardiness (Table 3, Fig. 3). Water content in progeny adult beetles was similar independent of cold treatments experienced by the parents (Table 3). On the other hand, cold treatment increased the glycerol content in insects with in average the three cold treatment together increasing glycerol levels by 32% in the next generation when compared to the 28°C treatment (Fig. 3A). Female beetles contain 24% more glycerol than males (Table 3). Insects that experienced colder temperatures had the lowest supercooling point (i.e. more negative temperatures) (Fig. 3B). We did not detect gender differences in supercooling point, although females had a slightly lower SCP than males (−13.57°C for females versus –12.85°C for males).

**Table 3:** Two-way ANOVAs for effects of temperature treatment, and gender on *O. communa* next generation adult water content, glycerol content, and supercooling point (SCP).

Response variable	Factor	Num df	F	*P*
Water content (%)	Gender (G)	1	0.13	0.7190
	Temperature (T)	3	0.62	0.6058
	G*T	3	0.04	0.9881
Glycerol content	G	1	325.71	<0.0001***
	T	3	122.94	<0.0001***
	G*T	3	40.77	<0.0001***
SCP	G	1	2.02	0.1648
	T	3	4.52	0.0094**
	G*T	3	0.11	0.9538

*** p<0.001,

** p<0.01,

* p<0.05,

°p<0.1.

Temperature treatments are 4, 6, 8, and 28°C, den. df = 16.

## Discussion

With the present study we show that previous generation cold experience impact survival, longevity, fecundity and physiological parameters in the progeny beetles. Particularly, adult’s experience of cold temperatures decreased progeny overall survival, but resulted in an increased fecundity and longevity in the next generation adults. Additionally, we observed increased cold hardiness in the progeny, which was explained by the beetles’ increased body glycerol content, and lower supercooling point.

Insects living in temperate regions have to cope with low winter temperatures, which may strongly influence the establishment and persistence of perennial populations in the field [37], [38], [58]. In general, survival rates of insects decrease with decreasing temperatures [23], [25], [27], [59], and this phenomenon has been also observed in *O. communa* [17], [23], [42]. Many insect species are thus able to increase their over-wintering survival through increased cold hardiness during the pre-winter months [31], [43], [60], [61], [62], [63]. However, previous studies have only reported responses of the insects’ current generation to adverse low temperatures [23], [25], [27], [59]. Whether low temperature experience of insects can stimulate increased tolerance to cold in the next generation remained until now an open question.

Additionally, at low temperatures, resources of the insect are diverted into high-quality egg production, which, due to physiological trade-offs, result in overall reduced fecundity [64]. Subsequently, because high quality eggs impose an increase in the metabolic rates, females may additionally experience reduced longevity [13], [14], [15], [65], [66], [67]. Because previous studies explored only the effects of low temperatures on one generation, positive physiological adaptation transmitted to the next generation remained hidden. Indeed, as we report here, higher longevity and increased cold hardiness may be seen as counterbalancing adaptations against reduced survival in the next generation.

Insects in nature have evolved various physiological mechanisms to improve their cold-hardiness, and hence their survival under cold environments [35], [39], [40], [74], [75], [76], [77], [78]. Indeed, cold acclimation often results in insects accumulating reserves of glycogen that are subsequently broken down into glycerol during cold temperatures in order to improve their cold-hardiness [31], [79], [80], [81], [82]. Additionally, in insects, glycerol accumulation is often associated with both lower SCP and reduced water content [32], [33], [34], [83]. Thus, insects can often improve their cold hardiness via physiological changes meditated by cold acclimation [28], [35], [36]. Our experiments showed that higher levels of glycerol content point in the progeny is mediated by maternal and paternal cold environment, in which, the coldest temperatures trigger females to allocate higher levels of glycerol in the progeny. This results in bettles to have lower supercooling points when parents experienced colder temperatures. It has been demonstrated that cold hardiness, via changes in the relative composition of their body fluids and fats, is a plastic trait that can be influenced by fluctuations in abiotic factors (e.g., temperature) throughout the breeding season of *O. communa* [37], [38]. The present experiment is in line with the idea that *O. communa* has strong environmentally-driven phenotypic plasticity to cold adaptation, and this can be transmitted to the next generation. Future work will need to assess whether increase in cold hardiness as shown here finally results in increased survival when next generation beetles are placed at low temperatures.

Transgenerational or maternal effects in animals are common, contribute to the complexity of phenotypes’ deployment in the progeny [2], [3], [4], [9], and are thought to have strong ecological and evolutionary significance [2], [3], [5], [4], [9]. For example, in *Drosophila melanogaster*, cold exposure associated with variation in feeding regimes resulted in fewer, smaller offspring, and resulted in a male-biased sex ratio [21], [22]. Similarly, A different nutritional experience resulted in modified offspring survival and fecundity in the gypsy moth, *Lymantria dispar* [84]. In our study, although cold temperatures have a little effect on progeny *O. communa* larval survival, progeny of females that experienced the coldest temperatures (4°C for 8 days) could lay more eggs compared to control.

Ultimately, explaining such results requires understanding by which mechanisms parent beetles’ experience of different environments can influence the physiology of their offsprings. In plants, it has recently been shown that small interfering RNAs and phytohormones are needed to mediated transgenerational priming for increased resistance against biotic and abiotic stresses [6], [85], [86]. Future work is thus necessary for measuring iRNAs-mediated transgenerational effects in beetles [87].

Independently of the mechanisms behind the observed results, we believe that previous generation environmentally-induced cold hardiness in *O. communa* populations may be a mechanism for insects tracking host-plant expansion into colder climates.

Life table analysis is a good appraisal tool for estimating the dynamics of insects’ populations [42], [54], [55], and has been widely applied for comparing the development and expansion potential of an insect population under various environmental conditions [42], [68], [69], [70], [71], [72], [73]. For instance, higher population expansion potential has been associated with higher intrinsic capacity for increase (*r*), higher net reproductive rate (*R_0_*), and higher finite rate of increase (*λ*) (42,68,73). Our results show that the intrinsic capacity for increase (*r*), the net reproductive rate (*R_0_*) and the finite rate of increase (*λ*) of *O. communa* progeny revealed the highest values after parents experienced the low temperature treatments of 4°C for 8 days. This implies that the descendants of *O. communa* can inherit the potential to foster population expansion when their parent experience low temperatures, such as when following the northward range expansion of ragweeds.

Such studies are thus not only needed to improve knowledge of fundamental physiological processes, but are also needed to further improve biological control practices of noxious and biodiversity-threatening weeds worldwide.
